# Pola-R-CHP or R-CHOEP for first-line therapy of younger patients with high-risk diffuse large B-cell lymphoma: a retrospective comparison of two randomized phase 3 trials

**DOI:** 10.1038/s41375-024-02420-6

**Published:** 2024-09-25

**Authors:** Georg Lenz, Hervé Tilly, Marita Ziepert, Bettina Altmann, Charles Herbaux, Fabian Frontzek, Maike Nickelsen, Calvin Lee, Jamie Hirata, Deniz Sahin, Saibah Chohan, Connie Lee Batlevi, Mark Yan, Franck Morschhauser, Norbert Schmitz

**Affiliations:** 1 Medical Department A, University Hospital Münster, Münster, Germany; 2 Centre Henri-Becquerel and University of Rouen, Rouen, France; 3 Institute for Medical Informatics, Statistics and Epidemiology, University Leipzig, Leipzig, Germany; 4 University of Montpellier, Montpellier, France; 5 Onkologie Lerchenfeld, Hamburg, Germany; 6 Genentech Inc., South San Francisco, CA USA; 7 F. Hoffmann-La Roche Ltd, Basel, Switzerland; 8 Hoffmann-La Roche Ltd, Mississauga, ON Canada; 9 University of Lille, Centre Hospitalier Universitaire, Lille, France

**Keywords:** B-cell lymphoma, B-cell lymphoma

## To the Editor:

Since development of R-CHOP for newly diagnosed diffuse large B-cell lymphoma (DLBCL) [1, 2], many attempts have been made to improve first-line therapy including studies which changed the timing/dosing of rituximab [3–5], substituted obinutuzumab for rituximab [6], added etoposide to CHOP in R-CHOEP [7], or changed to infusional regimens like dose-adjusted EPOCH-R [8]. While many studies combined targeted drugs with R-CHOP [9–11], Pola-R-CHP was the first to significantly improve progression-free survival (PFS) in patients with DLBCL aged 18–80 with an International Prognostic Index (IPI) 2–5. While overall survival (OS) was not significantly different, the reduction in relapses led to adoption of Pola-R-CHP as the new standard in many countries [12]. However, gaps in knowledge remain, in particular for younger, high-risk patients with DLBCL where R-CHOEP is a common regimen in Germany and the Scandinavian countries [13–15]. This study compares Pola-R-CHP and R-CHOEP using individual patient data from respective phase 3 studies.

## Methods

Study design, patient eligibility, randomization, endpoints, and statistical analyses of the two prospective, randomized phase 3 studies R-MegaCHOEP and POLARIX have been reported previously [7, 12]. POLARIX was an international, double-blind, placebo-controlled study, whereas R-MegaCHOEP was an open label study performed in Germany. Full inclusion and exclusion criteria of both studies were published previously [7, 12].

The current analysis compared outcomes of younger DLBCL patients (18–60 years) with age adjusted IPI (aaIPI) 2 or 3 treated with R-CHOEP on the R-MegaCHOEP study and patients treated with Pola-R-CHP on the POLARIX study. Protocol details are provided in Supplementary Data.

The current analysis limited the comparison to PFS and OS as the definition of event-free survival is variable. While positron-emission tomography in combination with computed tomography (PET/CT) was used to determine the response at end of treatment for patients treated in the POLARIX study, the response of patients treated with R-MegaCHOEP was determined by CT scan only. The incidence of adverse events occurring in both studies were also compared.

## Results

The study included 113 patients treated with Pola-R-CHP from the POLARIX study and 89 patients treated with R-CHOEP from the R-MegaCHOEP study. The majority of patients were male, with median ages of 52 and 51 for Pola-R-CHP and R-CHOEP groups, respectively. Major patient characteristics were similar across groups, including IPI factors and aaIPI (Table 1).

**Table 1:** Demographics and selected clinical characteristics at baseline.

	R-CHOEP	Pola-R-CHP
	*n* = 89	*n* = 113
Median age (range), years	51 (18–60)	52 (19–60)
Sex		
Female	32 (36%)	45 (39.8%)
Male	57 (64%)	68 (60.2%)
Abnormal LDH	86 (96.6%)	100 (88.5%)
ECOG		
0–1	61 (68.5%)	92 (81.4%)
>1	28 (31.5%)	21 (18.6%)
Stage		
I/II	1 (1.1%)	3 (2.7%)
III/IV	88 (98.9%)	110 (97.3%)
Presence of extranodal disease	67 (75.35%)	85 (75.2%)
aaIPI		
2	65 (73.0%)	99 (87.6%)
3	24 (27.0%)	14 (12.4%)
Presence of bulky disease ≥7.5 cm	51 (57.3%)	60 (53.1%)
Bone marrow disease	*n* = 89	*n* = 109
Yes	10 (11.2%)	26 (23.9%)
Cell of origin	*n* = 39	*n* = 87
ABC	9 (23.1%)	27 (31.0%)
GCB	24 (61.5%)	47 (54.0%)
Unclassified	6 (15.4%)	13 (14.9%)

*aaIPI* age-adjusted International Prognostic Index, *ABC* activated B-cell-like, *ECOG* Eastern Cooperative Oncology Group, *GCB* germinal center B-cell, *LDH* lactate dehydrogenase.

MYC and BCL2 rearrangements were available for 79.6% and 84.1% of Pola-R-CHP patients, and 42.7% and 48.3% of R-CHOEP patients. Translocation percentages were 8.9% and 17.9% in Pola-R-CHP, and 7.9% and 20.9% in R-CHOEP. Data on cell of origin were available for 87 Pola-R-CHP and 39 R-CHOEP patients (Table 1).

Median dose intensities of key drugs exceeded 98% in both groups (Supplementary Table 1). Radiotherapy was administered to 8 Pola-R-CHP and 21 R-CHOEP patients as part of the treatment protocol. Follow-up periods differed significantly, with a median of 28.1 months for Pola-R-CHP and 42.0 months for R-CHOEP.

**Fig. 1:**
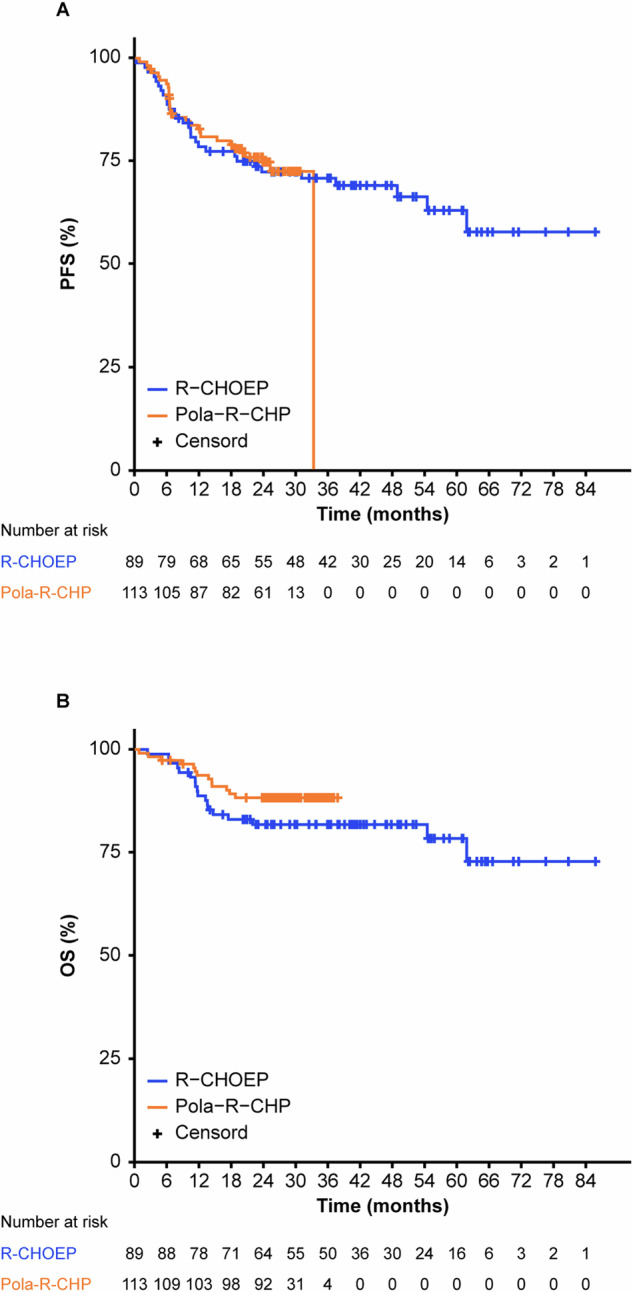
Survival of DLBCL patients treated with R-CHOEP or Pola-R-CHP respectively. Kaplan–Meier estimates of PFS (**A**) and OS (**B**). PFS at 2 years was 72.4% (95.0% CI 62.9%–81.8%) after R-CHOEP and 74.8% (95.0% CI 66.5%–83.0%) after Pola-R-CHP, respectively. The OS at 2 years was 81.7% (95.0% CI 73.6%–89.8%) after R-CHOEP and 88.3% (95.0% CI 82.3%–94.3%) after Pola-R-CHP. CI confidence interval, OS overall survival, PFS progression-free survival.

Two-year PFS was 74.8% for Pola-R-CHP and 72.4% for R-CHOEP, while OS was 88.3% for Pola-R-CHP and 81.7% for R-CHOEP (Fig. 1). Differences in efficacy were not significant. Separate evaluations for aaIPI 2 and 3 patients showed no significant differences in PFS and OS between the two treatments (Supplementary Figs. 1, 2).

PFS and OS were also analyzed for activated B-cell-like (ABC)- and germinal center B-cell (GCB)-subtypes of DLBCL. ABC-type patients treated with Pola-R-CHP showed higher 2-year PFS and OS rates compared with those treated with R-CHOEP (Supplementary Fig. 3). In GCB-type DLBCL, efficacy was similar between treatments.

R-CHOEP was associated with higher rates of leukocytopenia, infections, anemia, thrombocytopenia, and severe neuropathy compared to Pola-R-CHP. Low-grade toxicities affecting quality of life were also more frequent with R-CHOEP (Supplementary Table 2).

Mortality rates up to two years post-randomization were 13 for Pola-R-CHP and 16 for R-CHOEP. Disease progression or relapse was the main cause of death. Non-relapse mortality occurred in two Pola-R-CHP and three R-CHOEP patients. CNS relapses were noted in two Pola-R-CHP and three R-CHOEP patients.

## Discussion

Young high-risk (aaIPI 2–3) DLBCL patients remain difficult to treat although some improvement is noted with DA-EPOCH-R for IPI 3–5 patients and ibrutinib plus R-CHOP for younger patients with non-GCB DLBCL. A retrospective comparison of Pola-R-CHP from the POLARIX study and R-CHOEP from the R-MegaCHOEP trial shows comparable efficacy in terms of 2-year PFS and OS, though R-CHOEP has more associated toxicities. OS was particularly encouraging for patients with aaIPI 2 comprising about three quarters of all young high-risk patients treated with Pola-R-CHP or R-CHOEP [7, 8]. The remaining patients with aaIPI 3 showed promising efficacy, favoring Pola-R-CHP. Despite small patient numbers, which preclude further interpretation, an observed 13% difference in 2-year PFS and a 23% difference in OS both favoring Pola-R-CHP in the aaIPI 3 population was notable. A limitation of these comparisons was that PFS of patients treated with Pola-R-CHP was evaluated by PET while patients treated with R-CHOEP had CT scans only.

While efficacy was comparable between Pola-R-CHP and R-CHOEP we detected differences in acute toxicities. Patients with R-CHOEP experienced more cytopenias, infections and sensory neuropathies compared with patients treated with Pola-R-CHP. The less frequent acute toxicities underscore Pola-R-CHP as the regimen with a superior risk profile in this young high-risk population.

As the data arose from two randomized clinical trials with similar inclusion and exclusion criteria, the baseline characteristics and patient populations across the two studies are similar; therefore, propensity score matching was not performed for the cross-trial comparison. POLARIX and R-MegaCHOEP recruited patients during distinct epochs more than 10 years apart (2017–2019 and 2003–2009, respectively). Although the supportive care accompanying both regimens included the administration of granulocyte-colony stimulating factor, other prophylactic measures differed over time, and we cannot exclude that such differences influenced treatment-related morbidity and mortality. Recent therapeutic options including bispecific antibodies and chimeric antigen receptor T-cell therapy may have impacted OS in the more recently treated patients.

In conclusion, treatment outcomes of young, high-risk DLBCL patients were generally excellent with both R-CHOEP and Pola-R-CHP. Differences were not identified although Pola-R-CHP gave a promising signal in patients with aaIPI 3. Furthermore, the Pola-R-CHP regimen had a more favorable acute toxicity profile. The remaining few patients will hopefully benefit from targeted or immunotherapeutic approaches. To this end, the results of prospective studies incorporating novel substances and treatment modalities are eagerly awaited.

## Supplementary information

Supplemental data

## Data Availability

Data availability statement: For eligible studies, qualified researchers may request access to individual patient-level clinical data through the clinical study data request platform. At the time of writing this request, the platform is Vivli (https://vivli.org/ourmember/roche/). For up-to-date details on Roche’s Global Policy on the Sharing of Clinical Information and how to request access to related clinical study documents, see here: https://go.roche.com/data_sharing. Anonymized records for individual patients across more than one data source external to Roche cannot, and should not, be linked due to a potential increase in risk of patient re-identification.
