# Supplementary material for: Pola-R-CHP or R-CHOEP for first-line therapy of younger patients with high-risk diffuse large B-cell lymphoma: a retrospective comparison of two randomized phase 3 trials
Source: Leukemia. 2024 Sep 25;38(12):2709–11. doi: 10.1038/s41375-024-02420-6 (PMC11588652; doi:10.1038/s41375-024-02420-6)
Supplement: Supplementary file 1 — Supplemental data [file 41375_2024_2420_MOESM1_ESM.docx]

**SUPPLEMENTAL DATA**

**Pola-R-CHP or R-CHOEP for first-line therapy of younger patients with high-risk diffuse large B-cell lymphoma: a retrospective comparison of two randomized phase 3 trials**

Supplemental Methods:

R-CHOEP patients received eight cycles of CHOEP at 2-week intervals and six doses of rituximab. Central nervous system (CNS) prophylaxis and radiotherapy were included per investigator discretion. POLARIX patients received six 21-day cycles of R-CHOP or Pola-R-CHP, with CNS prophylaxis and radiotherapy at the investigator's discretion.

The primary endpoint of the R-MegaCHOEP study was investigator-assessed event-free survival (EFS), defined as time from randomization to disease progression, start of salvage treatment, additional unplanned treatment, relapse, or death from any cause. Key secondary endpoints were progression-free survival (PFS), defined as time from randomization to progression, relapse, or death from any cause, and overall survival (OS), defined as time from randomization to death from any cause as well as the cumulative incidences of relapse, secondary neoplasm, and different reasons of deaths. The primary end point of the POLARIX study was investigator-assessed PFS. Key secondary endpoints were OS and EFS where the latter was defined as investigator-assessed disease progression or relapse, death from any cause, initiation of any treatment for lymphoma that was not specified in the protocol including consolidative radiation without demonstrated relapse, or biopsy-confirmed residual disease after treatment completion.

Cell of origin and assessment of *BCL2*, *BCL6* and *MYC* translocation status, were performed at central laboratories in the POLARIX study and by reference pathology in the R-MegaCHOEP study. Molecular data were correlated with clinical outcome.

**Supplemental Table 1.** Dose delivery of R-CHOEP and Pola-R-CHP.

|  | **R-CHOEP** | **Pola-R-CHP** |
| --- | --- | --- |
| Relative dose of Rituximab (%) | 80 | 113 |
| Median (Min–Max) | 98.6 (16.9–109.8) | 100 (90.0–105.7) |
| Relative dose of Cyclophosphamide (%) | 81 | 113 |
| Median (Min–Max) | 98.3 (12.4–112.4) | 100 (85.8–105.7) |
| Relative dose of Doxorubicin (%) | 79 | 113 |
| Median (Min–Max) | 98.2 (12.4–109.2) | 100 (82.3–105.4) |
| Relative dose of Vincristine (%) | 78 | 0 |
| Median (Min–Max) | 100 (12.5-104.0) |  |
| Relative dose of Prednisone (%) | 78 | 113 |
| Median (Min–Max) | 100 (12.5–110.0) | 100 (58.3–103.5) |
| Relative dose of Etoposide (%) | 79 | 0 |
| Median (Min–Max) | 97.8 (12.4–112.2) |  |
| Relative dose of Polatuzumab vedotin (%) | 0 | 113 |
| Median (Min–Max) |  | 100 (71.6–111.2) |

The median relative dose intensities of rituximab, doxorubicin, and cyclophosphamide were greater than 98.0% in both treatment groups

Min, minimum; Max, maximum

**Supplemental Table 2.** Adverse events by high CTCAE Grade in the safety evaluable population from R-CHOEP and Pola-R-CHP with IPI 2–3 and ≤60 years. Patients treated with R-CHOEP experienced more leukocytopenia (Grade = 4 in 58.8% of patients) and more infections (Grade ≥3 in 30.7% of patients) than patients treated with Pola-R-CHP (15.6% and 14.7%). Anemia and thrombocytopenia were also more frequent after R-CHOEP than after Pola-R-CHP. More patients developed severe neuropathy following R-CHOEP compared with Pola-R-CHP-treated patients (7.1% vs. 0.0%). Similarly, low grade toxicities such as constipation, diarrhea, mucositis, nausea, and vomiting, which may impede quality of life during treatment, were more frequent in patients treated with R-CHOEP.

|  | **R-CHOEP** | **POLA-R-CHP** |
| --- | --- | --- |
|  | *n = 89* | *n = 109* |
| Hematological | | |
| Leukocytopenia |  |  |
| Grade 4 | 30/51 (58.8%) | 17/109 (15.6%) |
| Thrombocytopenia |  |  |
| Grade ≥3 | 15/51 (29.4%) | 2/109 (1.8%) |
| Anemia |  |  |
| Grade ≥3 | 36/84 (42.9%) | 11/109 (10.1%) |
| Non-hematological | | |
| Cardiac |  |  |
| Grade 1–2 | 3/82 (3.7%) | 2/109 (1.8%) |
| Grade ≥3 | 1/82 (1.2%) | 0/109 (0.0%) |
| Constipation |  |  |
| Grade 1–2 | 22/83 (26.5%) | 28/109 (25.7%) |
| Grade ≥3 | 3/83 (3.6%) | 0/109 (0.0%) |
| Diarrhea |  |  |
| Grade 1–2 | 28/82 (34.1%) | 32/109 (29.4%) |
| Grade ≥3 | 2/82 (2.4%) | 2/109 (1.8%) |
| Infections |  |  |
| Grade 1–2 | 28/88 (31.8%) | 32/109 (29.4%) |
| Grade ≥3 | 27/88 (30.7%) | 16/109 (14.7%) |
| Mucositis |  |  |
| Grade 1–2 | 27/81 (33.3%) | 27/109 (24.8%) |
| Grade ≥3 | 5/81 (6.2%) | 2/109 (1.8%) |
| Nausea |  |  |
| Grade 1–2 | 41/82 (50.0%) | 53/109 (48.6%) |
| Grade ≥3 | 0/82 (0.0%) | 3/109 (2.8%) |
| Sensory neuropathy |  |  |
| Grade 1–2 | 45/85 (52.9%) | 55/109 (50.5%) |
| Grade ≥3 | 6/85 (7.1%) | 0/109 (0.0%) |
| Vomiting |  |  |
| Grade 1–2 | 21/81 (25.9%) | 16/109 (14.7%) |
| Grade ≥3 | 1/81 (1.2%) | 2/109 (1.8%) |


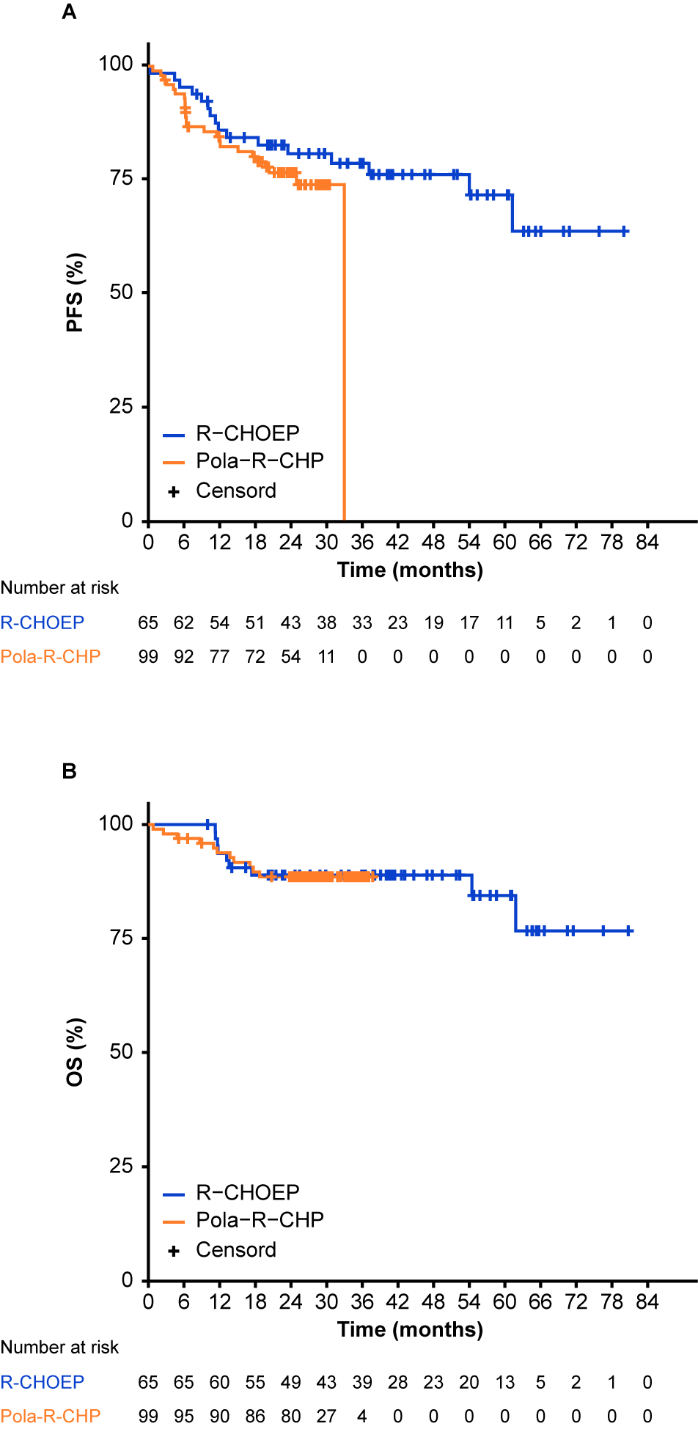


**Supplemental Fig. 1 Kaplan–Meier Estimates of PFS (A) and OS (B) in aaIPI 2 patients.**  In patients with aaIPI 2, PFS at 2 years was 80.8% (95.0% CI 71.0%–90.6%) after R-CHOEP and 76.6% (95.0% CI 68.0%–85.2%) after Pola-R-CHP, respectively. The OS was 89.0% (95.0% CI 81.3%–96.7%) after R-CHOEP and 88.6% (95.0% CI 82.3%–95.0%) after Pola-R-CHP

aaIPI, age-adjusted International Prognostic Index; CI, confidence interval; OS, overall survival; PFS, progression-free survival


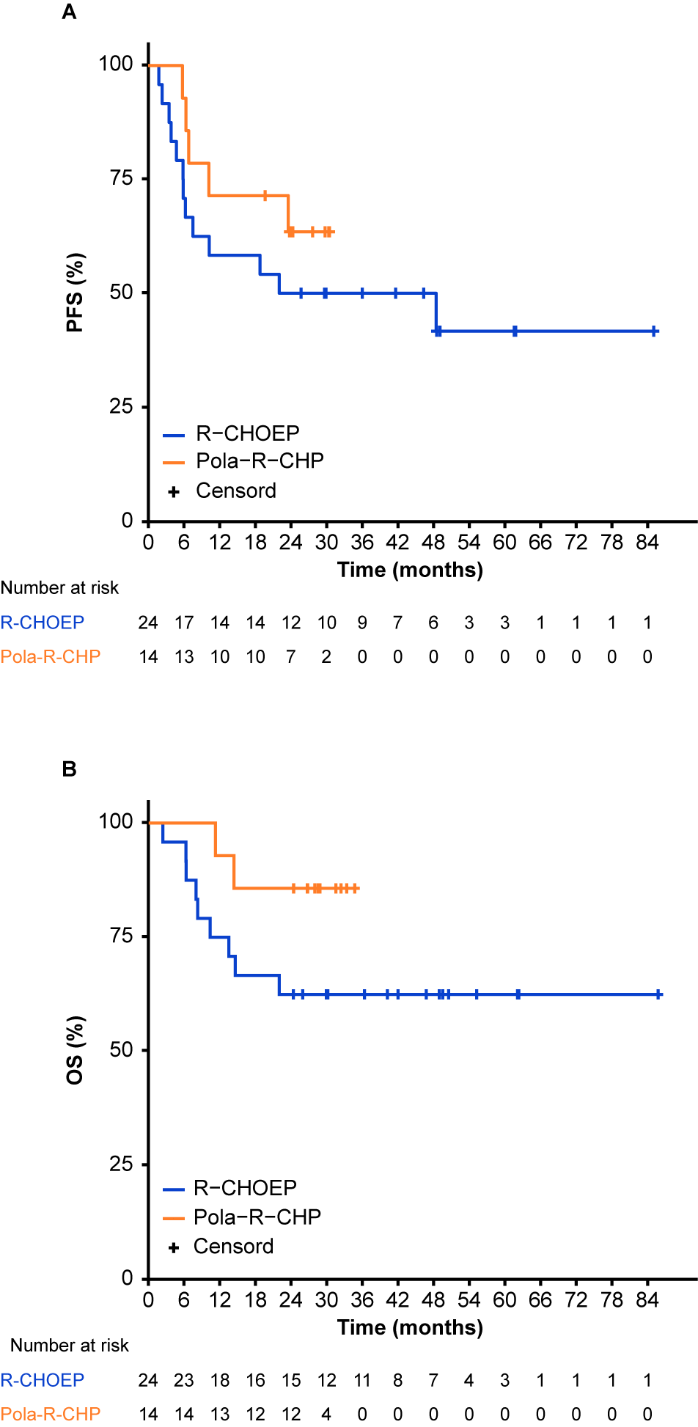


**Supplemental Fig. 2. Kaplan–Meier Estimates of PFS (A) and OS (B) in aaIPI 3 patients.**  In patients with aaIPI 3, the PFS at 2 years was 50.0% (95.0% CI 30.0%–70.0%) after R-CHOEP (n = 24) and 63.5% (95.0% CI 37.9%–89.1%) after Pola-R-CHP (n = 14) (Fig 2A). The OS of patients with aaIPI 3 treated with R-CHOEP and Pola-R-CHP were 62.5% (95.0% CI 43.1%–81.9%) and 85.7% (95.0% CI 67.4%–100.0%) (Fig. 2B). Neither PFS nor OS significantly differ between treatment groups when patients with aaIPI 2 or aaIPI 3 were analyzed separately.

aaIPI, age-adjusted International Prognostic Index; CI, confidence interval; OS, overall survival; PFS, progression-free survival


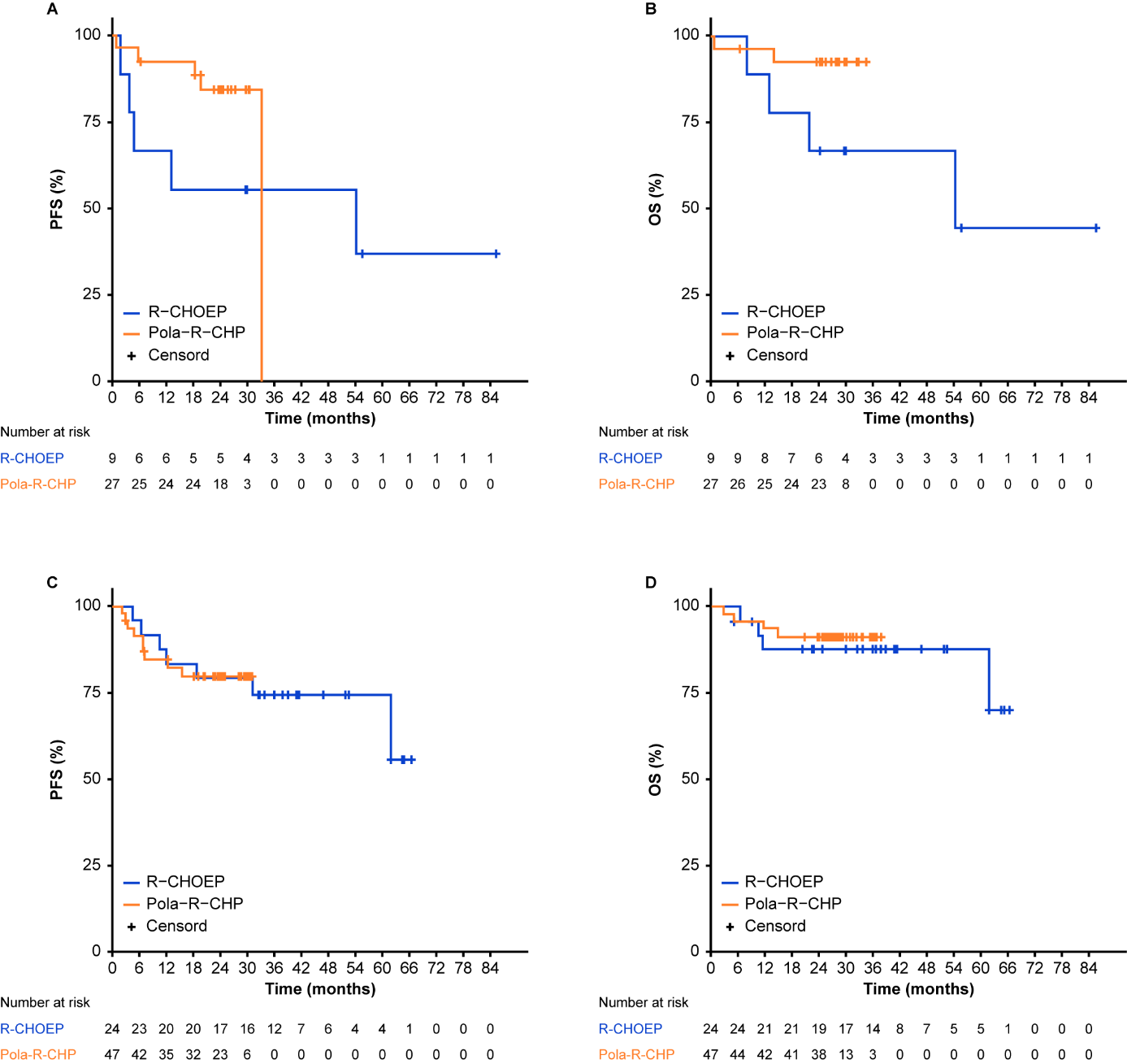


**Supplemental Fig. 3. Kaplan–Meier Estimates of Efficacy End Points in COO. ABC PFS (A), OS (B); GCB PFS (C), OS (D).** Evaluating PFS and OS of patients with ABC- (Fig. 3A, B) or GCB-subtype (Fig. 3C, D) of DLBCL identified no significant differences when patients were treated with R-CHOEP or Pola-R-CHP. PFS of patients with ABC-type DLBCL treated with Pola-R-CHP was higher than PFS of patients treated with R-CHOEP demonstrating 2-year PFS rate 84.5% (95.0% CI 70.5%–98.5%) and 2-year OS rate 92.4% (95.0% CI 82.4%–100.0%) in ABC-type DLBCL treated with Pola-R-CHP. Patients with GCB-type DLBCL treated with Pola-R-CHP showed 2-year PFS rate 79.8% (95.0% CI 67.9%–91.6%) and 2-year OS rate 91.3% (95.0% CI 83.1%–99.5%). In ABC-type DLBCL, PFS and OS was generally higher with Pola-R-CHP therapy. In GCB-type DLBCL, PFS and OS was similar between Pola-R-CHP or R-CHOEP. Patient numbers were small and confidence intervals were wide; therefore, the analysis warrants further evaluation

aaIPI, age-adjusted International Prognostic Index; ABC, activated B-cell-like; COO, cell of origin; CI, confidence interval; DLBCL, diffuse large B-cell lymphoma; GCB, germinal center B-cell; OS, overall survival; PFS, progression-free survival
